# Continuous and intermittent theta burst stimulation of primary visual cortex do not modulate resting state functional connectivity: A sham‐controlled multi‐echo fMRI study

**DOI:** 10.1002/brb3.2989

**Published:** 2023-04-16

**Authors:** Remy Cohan, Sara A. Rafique, Karlene S. Stoby, Diana J. Gorbet, Jennifer K. E Steeves

**Affiliations:** ^1^ Centre for Vision Research Faculty of Health and Department of Psychology, York University Toronto Ontario Canada; ^2^ York MRI Facility York University Toronto Ontario Canada

**Keywords:** functional connectivity, multiecho fMRI, resting state, theta burst stimulation (TBS), transcranial magnetic stimulation (TMS), visual networks

## Abstract

**Introduction**: Theta burst stimulation (TBS) is a type of rTMS protocol which has the advantage of a shorter delivery time over traditional rTMS. When applied to motor cortex, intermittent TBS (iTBS) has been shown to yield excitatory aftereffects, whereas continuous TBS (cTBS) may lead to inhibitory aftereffects, both lasting from minutes to hours. The majority of TBS research has targeted motor, frontal, and parietal regions, and to date very few studies have examined its efficacy at visual areas. We designed a sham‐controlled study to investigate the immediate poststimulation and short‐term (1 h post‐stimulation) effects of iTBS and cTBS to V1.

**Methods**: Using multiecho functional magnetic resonance imaging, we measured the direct and indirect effects of TBS by comparing resting state functional connectivity (FC) before and after stimulation in whole brain networks, and seeds from V1 (stimulation site) and neighboring occipital and parietal visual networks. In addition, we also measured pre‐ and post‐TBS phosphene thresholds (PTs) to examine the modulatory effects of TBS on cortical excitability.

**Results**: We found no changes in FC for iTBS, cTBS or sham stimulation conditions from baseline to poststimulation timepoints. Additionally, cTBS and iTBS had no effect on visual cortical excitability.

**Conclusion**s: Our results indicate that unlike our previous low frequency rTMS to V1 study, which resulted in widespread FC changes up to at least 1 h after stimulation, TBS to V1 does not affect FC. Contrary to the studies showing comparable TBS and rTMS aftereffects in motor and frontal regions, our findings suggest that a single session of cTBS or iTBS to V1 at 80% PT using a standard protocol of 600 pulses may not be effective in targeting FC, especially in clinical settings where therapy for pathological networks is the goal.

## Graphical abstract

We investigated the short term (up to 1 hr) effects of continuous and intermittent theta burst stimulation (TBS) to primary visual cortex on resting state functional connectivity (FC) of focal visual networks and remote brain regions. Our findings suggest that a single session of TBS to V1 does not appear to produce significant short‐term alterations in functional connectivity following stimulation.

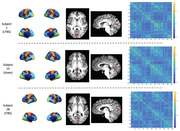

## 1 INTRODUCTION

Since the advent of noninvasive neuromodulation, transcranial magnetic stimulation (TMS) has proven to be a powerful tool for inducing transient alteration of neural activity and has allowed for causal mapping of nodes within neural networks (Barker et al., 1985; Day et al., 1989; Kobayashi & Pascual‐Leone, 2003; Rafique et al., 2015; Solomon‐Harris et al., 2016). Commonly employed TMS protocols such as low (1 Hz) and high (10 Hz) frequency repetitive TMS (rTMS) have been shown to alter focal neural activity at the stimulation site as well as remote neural networks with effects lasting from minutes to days (Fox et al., 2012; Pascual‐Leone et al., 2000; Rafique & Steeves, 2022; Rafique et al., 2016). Although the mechanism of these effects are not fully understood, these changes have been attributed to long‐term potentiation (LTP) and long‐term depression (LTD) involved in synaptic plasticity (Barker et al., 1985; Bliss & Lomo, 1973; Day et al., 1989).

rTMS has shown effectiveness both as a research tool and a treatment modality in neurological and neuropsychiatric disorders (Mi et al., 2020; Rafique et al., 2016). The quest for shorter stimulation time and lasting aftereffects however has led to a modified variation of rTMS, namely theta burst stimulation (Hill, 1978; Huang et al., 2011; Larson et al., 1986). Since its inception, research employing TBS protocols have shown that modifying traditional stimulation parameters (i.e., frequency, intensity, pattern and duration) can lead to focal dissociable inhibitory and excitatory effects (Gilio et al., 2007; Hess & Donoghue, 1996). These effects, however, have been mostly examined in primary motor cortex (M1) via motor evoked potentials (MEPs) measured using electromyography (EMG) to evaluate the efficacy of stimulation and to determine optimal stimulation intensity levels at M1 (Huang et al., 2011; Rossini & Rossi, 1998). For example, Huang et al. (2005) previously showed that the application of continuous TBS (cTBS; 40 s trains of uninterrupted TBS) to M1 lowered the amplitude of MEPs, while the application of intermittent TBS (iTBS; 2s trains of TBS repeated every 10 s) to M1 increased the amplitude of MEPs with aftereffects lasting up to 60 min.

With a growing number of confirmatory studies, TBS has gained a foothold in research and clinical settings mainly due to its shorter (∼ 3 min) delivery time over rTMS (∼28 min), which can drastically improve efficiency of empirical research in the lab and patient compliance in clinical settings. Nevertheless, it is assumed that for any TMS‐based protocol to be considered an effective treatment, its therapeutic effects should last long enough to induce measurable changes at the neural and behavioral levels. Thus far, TBS has proven to be effective in treating depression, and has shown promising results in neurorehabilitation and chronic pain (Blumberger et al., 2018; Moisset et al., 2015; Talelli et al., 2007). However, the neural underpinnings of TBS aftereffects beyond the stimulation time remains and open area of research. Moreover, the efficacy of TBS at the visual cortex and, therefore, its value and use in visual disorders is lacking. Pilot data in the therapeutic effects of occipital (and cerebellar) TBS in ameliorating symptoms of patients with Mal de Débarquement syndrome (Cha et al., 2019) has been explored and shown promising results. This patient population suffers from the chronic phantom perception of oscillating vertigo thought to be caused by changes in neural excitability in the balance system, in the absence of movement or vestibular and ocular inputs (Van Ombergen et al., 2016). Both cTBS and iTBS have been explored in phantom limb sensation after spinal cord injury, and cTBS proved effective in suppressing phantom sensations (Nardone et al., 2016). TBS is also a potentially viable candidate as an investigative tool to study the etiology of visual disorders, including cortical blindness and poststroke or postenucleation visual hallucination such as Charles Bonnet syndrome (CBS; Cox & ffytche, 2014; Gothe et al., 2002; Wen et al., 2018) and phosphenes (Rafique et al., 2018), and to develop neuromodulation based therapies for these conditions in the future.

To probe TMS effects at the neuronal level, neuroimaging techniques such as resting state fMRI (rs‐fMRI) can measure whole brain and regional changes in connectivity of stimulated targets, respectively. Using blood oxygenation level dependent (BOLD) imaging, rs‐fMRI is an indirect measure of physiological dependencies between different anatomical locations determined through various functional connectivity (FC) data analysis techniques (Biswal et al., 1995; Fox et al., 2005; Friston, 1994). rs‐fMRI can track changes in the brain's networks both in health and disease, for example previous studies have demonstrated distinct alterations in visual networks and the default mode network (DMN) of patients with strabismus and amblyopia (Peng et al., 2021; Shao et al., 2019), late blindness (Wen et al., 2018) and CBS (ffytche et al., 1998). Additionally, studies using rs‐fMRI investigating the inhibitory and excitatory effects of cTBS and iTBS to motor, parietal and frontal brain regions have shown that these protocols modulate opposite connectivity patterns in focal and remote brain areas (Cocchi et al., 2015; de Wandel et al., 2020; Gratton et al., 2013). To date, however, only one rs‐fMRI study investigated the effects TBS to primary visual cortex (V1; Rahnev et al., 2013), and a few neuroimaging studies have examined the effects of TBS to specific nodes within the visual network (in areas beyond V1), for example, at the occipital cortex in visual category‐selective areas to measure changes at the stimulation site and FC between different vision‐related cortical and subcortical regions before and after TBS (Groen et al., 2021; Handwerker et al., 2020; Lasagna et al., 2021; Rahnev et al., 2013).

We previously examined the effects of a single session of 1 Hz rTMS to V1 using MRI‐guided neuronavigation and rs‐fMRI to determine immediate and short‐term effects of stimulation and found no immediate effects on FC following a single 20‐min session of 1 Hz rTMS but widespread changes in FC were observed at 1 h following stimulation (Rafique & Steeves, 2022). To determine whether TBS offers a shorter protocol with equivalent effects compared to traditional TMS at V1, we similarly examined the immediate and short‐term (up to 1 h post‐TBS) effects of cTBS and iTBS to V1 on whole brain FC as well as nodes in occipital and parietal visual networks using MRI‐guided neuronavigation. Parietal areas, such as the precuneus cortex were chosen mainly due to their interconnectivity with occipital visual areas and their involvement in resting state networks such as the DMN (Fox et al., 2005; Raichle, 2011; Zhang et al., 2018). Additionally, in line with a study done by Franca et al. (2006) showing changes in excitability thresholds measured via phosphene thresholds (PTs) post‐TBS (Franca et al., 2006), we also set out to determine TBS aftereffects on PTs by comparing baseline and 1 h post‐TBS values.

## 2 METHODS AND MATERIALS

### 2.1 Participants

Thirty‐one right‐handed participants (14 males and 17 females, *M*_age_ = 23 *SD* = 4 years) with no history of medical, neurological, or psychological disorders and no contradictions to TMS and MRI consented to participate. Participants had normal or corrected‐to‐normal vision and underwent screening including eligibility questionnaires, vision, and cognitive assessments. Data from one participant was omitted due to high motion artifacts detected during image preprocessing.

### 2.2 Experimental overview

**FIGURE 1:**
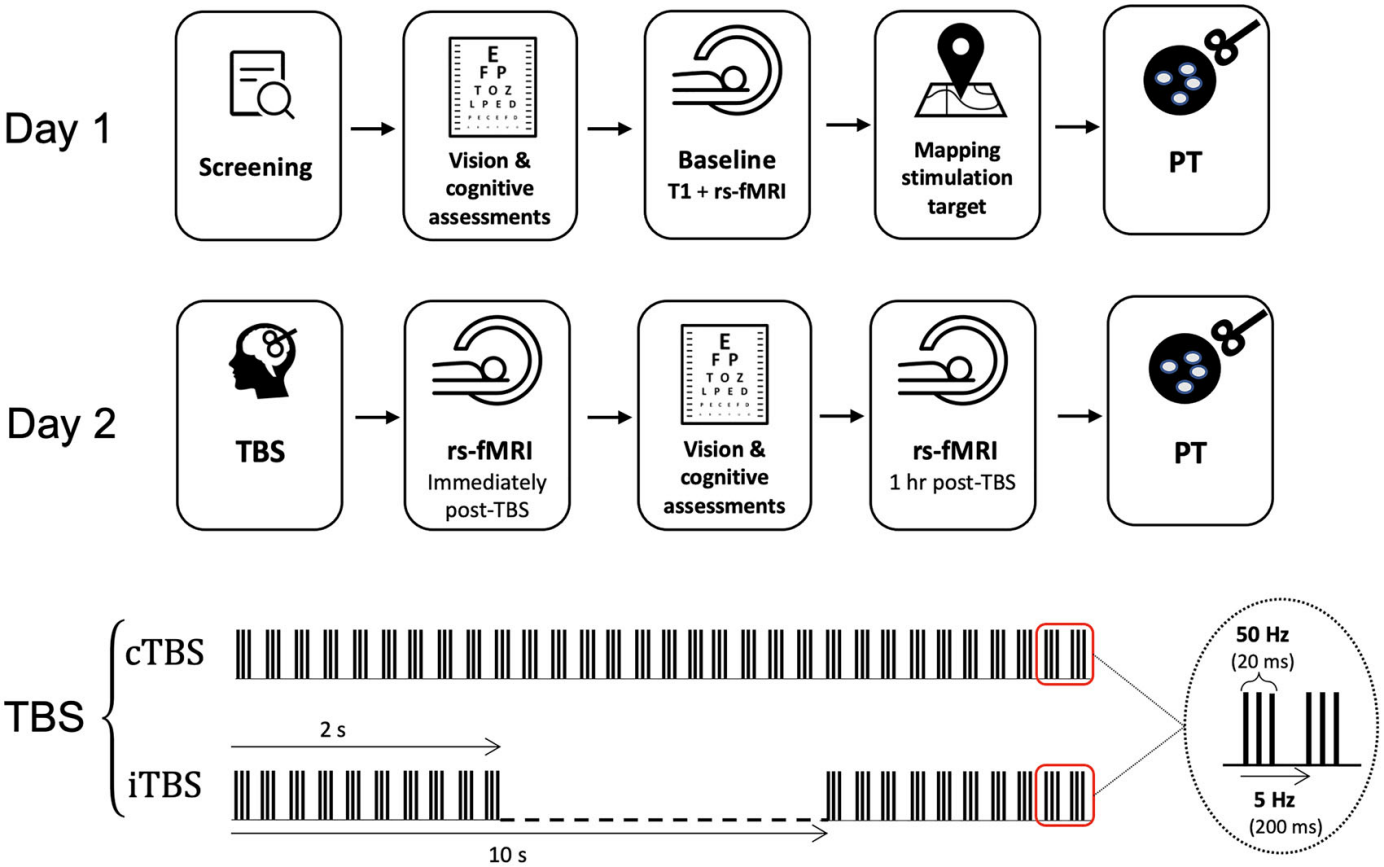
An overview of the experimental design and TBS parameters. Arrows between windows indicate the order of events. rs‐fMRI = resting state functional magnetic resonance imaging, MoCA = Montreal cognitive assessment, TBS = theta burst stimulation, iTBS = intermittent TBS; 2s trains of TBS repeated every 10 s, cTBS = continuous TBS; 40 s trains of uninterrupted TBS, PT = phosphene threshold.

This study was approved by the Office of Research Ethics at York University and took place over two sessions separated by one week. In a pseudo random fashion, and naïve to TMS, participants were assigned to one of three conditions: cTBS, iTBS, or sham. On day 1, at approximately 1 pm, each participant completed the screening, including eligibility questionnaires, vision assessment and the Montreal Cognitive Assessment (MoCA) versions 7.1‐7.3 (Nasreddine et al., 2005). At approximately 1:30 pm, baseline anatomical MRI and rs‐fMRI were obtained, and subsequently PTs were measured. In order to prevent residual effects from PT and to minimize diurnal effects, participants were tested one week following the baseline session at approximately the same time of the day. On day 2, participants underwent TBS and poststimulation MRI scans were acquired at two different timepoints—immediately following TBS (within 5 min) and 1 h after TBS. PTs were then remeasured after scans were completed (see Figure 1 for the experimental overview).

### 2.3 Vision and cognitive assessments

All participants were required to complete and pass three basic visual assessments for eligibility for normal or corrected‐to‐normal vision (>0.04 LogMAR; stereoacuity ≥ 50″, normal color vision). Monocular and binocular visual acuities were measured using the standardized ETDRS LogMAR vision chart (precision Vision, La Salle, IL), stereo acuity was measured using the Titmus Stereoacuity test (Stereo Optical Company Inc., Chicago, IL), and color vision was assessed using the Ishihara Colour Plates (Kanehara Trading Inc., Tokyo, Japan).

Participants were also required to complete and pass the MoCA (v7.1‐7.3). The MoCA is a screening tool that detects cognitive impairment with scores ranging from 0 to 30. It evaluates attention, concentration, and executive function (Nasreddine et al., 2005). The inclusion cut‐off was set at scores equal and greater than 26 (Yeung et al., 2020). All participants were able to meet the cut‐off. Statistical analyses for visual acuity and MoCA scores were performed using R statistical software (v 3.3.3; R Foundation for Statistical Computing, Vienna, Austria; www.R‐project.org) and the *lmer* package for multilevel modeling (Kuznetsova et al., 2017).

### 2.4 PT measurement

A phosphene is the experience of light in the absence of visual stimuli. PT is a measure of visual cortex excitability that is accomplished by stimulation of visual cortex leading to a subjective percept of light in participant's visual field. Visual cortex excitability thresholds can vary greatly across individuals (Stewart et al., 2001) presumably reflecting individual cortical excitability. As such, PTs can be used to determine appropriate individual stimulation intensity for TMS administration at the visual cortex in the same way that motor threshold (MT) is used to determine TMS intensity when applied to the motor cortex. Phosphenes are elicited when stimulation is applied from 1−5 cm above the inion and 0−3 cm laterally, in either hemisphere being tested (Elkin‐Frankston et al., 2010). Participants sat in a dimly lit room while wearing a blindfold with eyes closed. Four locations including the inion, 2 cm above the inion, 2 cm to the left of the inion, and 2 cm above the 2 cm to the left of the inion marker were identified as the stimulation grid. Using single‐pulse TMS with the coil center held tangential to the scalp and handle orientated 90^0^ laterally to the midline, individual PTs were measured for each subject. The minimum stimulator output intensity was set at 50%, and 10 pulses were delivered to the marker 2 cm above the inion. Each pulse was 6 s apart. Upon delivery of a single TMS pulse, participants were instructed to respond “yes/no/maybe” corresponding to whether a phosphene was perceived. At each location, the stimulator output was increased in 5% increments until a phosphene was evoked. For safety, we limited the maximum output setting to 90% intensity (Wassermann, 1998). If no phosphenes were evoked after 10 pulses, the coil was moved to a new position in the stimulation grid until the participant responded “yes,” which was then marked as the hotspot. Subsequently, at the hotspot, the threshold was modified by 1% increments to refine the PT. A threshold was defined as the intensity at which 50% of pulses (5/10 pulses) resulted in a “yes” response. The blindfold was removed every 10–15 min, when necessary, for a minimum of 3–5 min, to prevent dark adaption (Boroojerdi et al., 2000). PTs were analyzed using R statistical software (v 3.3.3; R Foundation for Statistical Computing, Vienna, Austria; www.R‐project.org) and the *lmer* package for multilevel modeling.

### 2.5 TBS

Participants underwent one of the three TBS stimulation conditions (cTBS, iTBS, or sham). TMS was delivered with a Magstim Rapid ^2^ Plus 1 stimulator and an air‐cooled figure‐of‐eight stimulation coil (Magstim, Whiteland, Wales, UK). Participants were stimulated at 80% individual PT that was initially determined on day 1. The cTBS protocol consisted of bursts containing three pulses at 50 Hz with a 20 ms inter‐stimulus interval (ISI) repeated at 5 Hz intervals with 200 ms ISI, applied continuously for 40 s, providing a total of 600 pulses (Huang et al., 2005). The iTBS protocol consisted of the same bursts containing three pulses at 50 Hz, repeated at 5 Hz intervals, however applied in 2 s trains repeated every 10 s for a total of 190 s, providing a total of 600 pulses (Huang et al., 2005). The sham TBS protocol was the same as the active conditions, except it was performed using the placebo sham coil. Four participants received sham iTBS and six received sham cTBS. The sham coil is equipped with a shield that attenuates the magnetic field while mimicking auditory and stimulation effects of an active coil.

TMS was delivered using Brainsight's neuronavigation system to ensure the accuracy of the coil position throughout stimulation (Rogue Research, Montreal, QC, Canada). Participants’ anatomical MR images were reconstructed and coregistered to their three‐dimensional cortical surfaces in Brainsight. The stimulation site corresponded to the volume of interest (VOI) in V1 in our previously published magnetic resonance spectroscopy (MRS) study of the same cohort (Stoby et al., 2022). The stimulation site was mapped on each participant's corresponding anatomical image in Brainsight by manually matching the anatomical landmarks to the center of the MRS VOI images. The neuronavigation system precisely maps individually targeted stimulation sites and accounts for anatomical variability across participants. We used the same coil both for determining phosphene thresholds and for TBS, however, for TBS the coil was held parallel to the midline with the handle pointing downwards and the coil center tangential to the head to minimize coil to cortex distance. This coil orientation was necessary due to the fact that we stimulated the calcarine sulcus (V1) by placing the center of the coil 1–2 cm around the center of the inion. The exact stimulation location differed for each participant due to individual anatomical differences observed on T1 images in Brainsight neuronavigation system. Participants sat upright with their eyes open, and their chin stabilized by a chin rest, and while TBS was delivered with the coil placed 2 cm above the center of the inion, PTs were measured at varying locations around the inion (2 cm radius).

### 2.6 MRI

Both anatomical and functional sequences were obtained at baseline, immediately post‐TBS, and 1 h post‐TBS using a 3 Tesla Siemens Magnetom Prisma magnetic resonance scanner with a 32‐channel high‐resolution array head coil (Siemens, Erlangen, Germany). Participants were instructed to remain motionless with their eyes closed while refraining from falling asleep.

Anatomical high‐resolution T‐weighted magnetization‐prepared rapid gradient echo (MPRAGE) sequence was acquired first [number of slices = 192, in‐plane resolution = 1 mm × 1 mm, slice thickness = 1 mm, imaging matrix = 256 × 256, repetition time (TR) = 2300 ms, echo time (TE) = 2.26 ms, inversion time (TI) = 900 ms, flip angle = 8°, field of view (FoV) = 256 mm, acquisition time = approximately 5 min]. Resting state functional imaging was acquired with T2* weighted whole brain echo planar ME imaging (number of contiguous axial slices = 43; in‐plane resolution = 3.4 × 3.4 mm; slice thickness = 3 mm; imaging matrix = 64 × 64; TR = 3000 ms; TE_1_ = 14.0 ms, TE_2_ = 30.08 ms, TE_3_ = 46.16 ms; flip angle = 83°; FoV = 216 mm; acquisition time = 10 min).

### 2.7 fMRI data preprocessing

Preprocessing and denoising were performed in AFNI (Cox & Hyde, 1997) using multiecho independent component analysis (ME‐ICA, v3.2). ME‐ICA uses the TE‐dependence of the BOLD signal to separate true BOLD signal from non‐TE‐dependent fluctuations that result from sources of noise (Kundu et al., 2012). Prior to denoizing with ME‐ICA, data preprocessing steps included discarding the first five volumes of each resting state fMRI time‐series. Images were skull‐stripped, and image intensity was normalized (3dSkullStrip). The functional images were de‐obliqued (3dWarp). Large signal transients were removed via interpolation (“despiking,” 3dDespike) and slice time correction was applied (3dTshift). Motion correction parameters were calculated using the middle echo (TE_2_ = 30.08 ms, 3dvolreg). Skull‐stripped anatomical and functional images were coregistered by registering the middle echo image from the first timepoint to the anatomical image using an affine alignment procedure with the local Pearson's correlation and T2* weights (3dAllineate). Anatomical and functional images were kept in native space. After the three TEs were optimally combined, ME‐ICA denoising was applied. BOLD signal was identified as independent components having linearly TE‐dependent percentage signal changes. Non‐BOLD noise components were removed from the time‐series by ME‐ICA using linear regression. The output of this process included a functional time‐series reconstructed to include only the BOLD signal components of the data. This preprocessed and denoised time‐series was used in all subsequent stages of the data analysis. Subject‐ and session‐specific quality checks were performed after preprocessing and denoising by inspecting plots of estimated head motion and anatomical‐functional alignment. In addition, inclusion of subjects in further stages of analysis required the identification of at least 10 BOLD‐like components by ME‐ICA. Images from one subject were omitted due to excessive motion and few detected BOLD‐like components.

### 2.8 Functional connectivity analyses

To analyze the rs‐fMRI data, we used two different approaches: (1) volumetric seed‐based FC analysis in MNI space and (2) surface‐based FC analysis with individual parcellation. For each analysis, an exploratory stepwise approach was used to first determine group‐level whole brain seed‐to‐voxel connectivity profiles followed by ROI‐to‐ROI and seed‐to‐target analyses in occipital and parietal visual areas to probe group differences at different timepoints. Given the individual variability in response to stimulation and statistical stringencies involved in exploratory analyses, this approach was deemed critical in order to detect TBS aftereffects at the connectome level.

#### 2.8.1 Analysis 1: Volumetric seed‐based connectivity analysis in MNI space

For this analysis we used MATLAB R2019a (MathWorks, Natick, MA, USA), and CONN Toolbox v20.b (www.nitrc.org/projects/conn). The preprocessed and denoised rs‐fMRI images and the preprocessed anatomical scans for each subject and each session were input into CONN. Functional and anatomical scans were normalized into standard MNI space and segmented into grey matter (GM), white matter (WM) and cerebrospinal fluid (CSF) tissue classes using SPM12 unified segmentation and normalization procedure (Ashburner & Friston, 2005). Functional images were then spatially smoothed by a 6 mm Gaussian kernel of full width at half‐maximum. Using an anatomical component‐based noise correction (CompCor) five principal components from CSF, GM, and WM were extracted (Behzadi et al., 2007) and confound regression was performed via principal component analysis (PCA) in order to remove non‐BOLD signals (Power et al., 2014).

Using an exploratory whole brain seed‐based connectivity (SBC) approach, subject‐specific cross‐correlation matrices between the seed and the whole brain, as well as non‐BOLD confounds were fitted to a first‐level model (Whitfield‐Gabrieli & Nieto‐Castanon, 2012). We explored SBC in two seeds, the stimulation site and the precuneus cortex based on the Harvard‐Oxford atlas coordinates (Desikan et al., 2006). For the stimulation site, a 10 mm spherical seed ROI was centered at the average stimulation site coordinates (*x* = 1, *y* = −72, *z* = 13). The subject‐specific standardized stimulation site coordinates were extracted following manual coregistration of individual anatomical scans into MNI in Brainsight neuronavigation system using the anterior commissure (AC)‐posterior commissure (PC) technique. SBC maps were computed as the Fisher‐transformed bivariate correlation coefficients between the seeds’ time‐series and each individual voxel time‐series. Given the nonnormal nature of the data, nonparametric (permutation‐randomization with 1000 simulations) FDR‐corrected statistics were chosen for further analyses.

**FIGURE 2:**
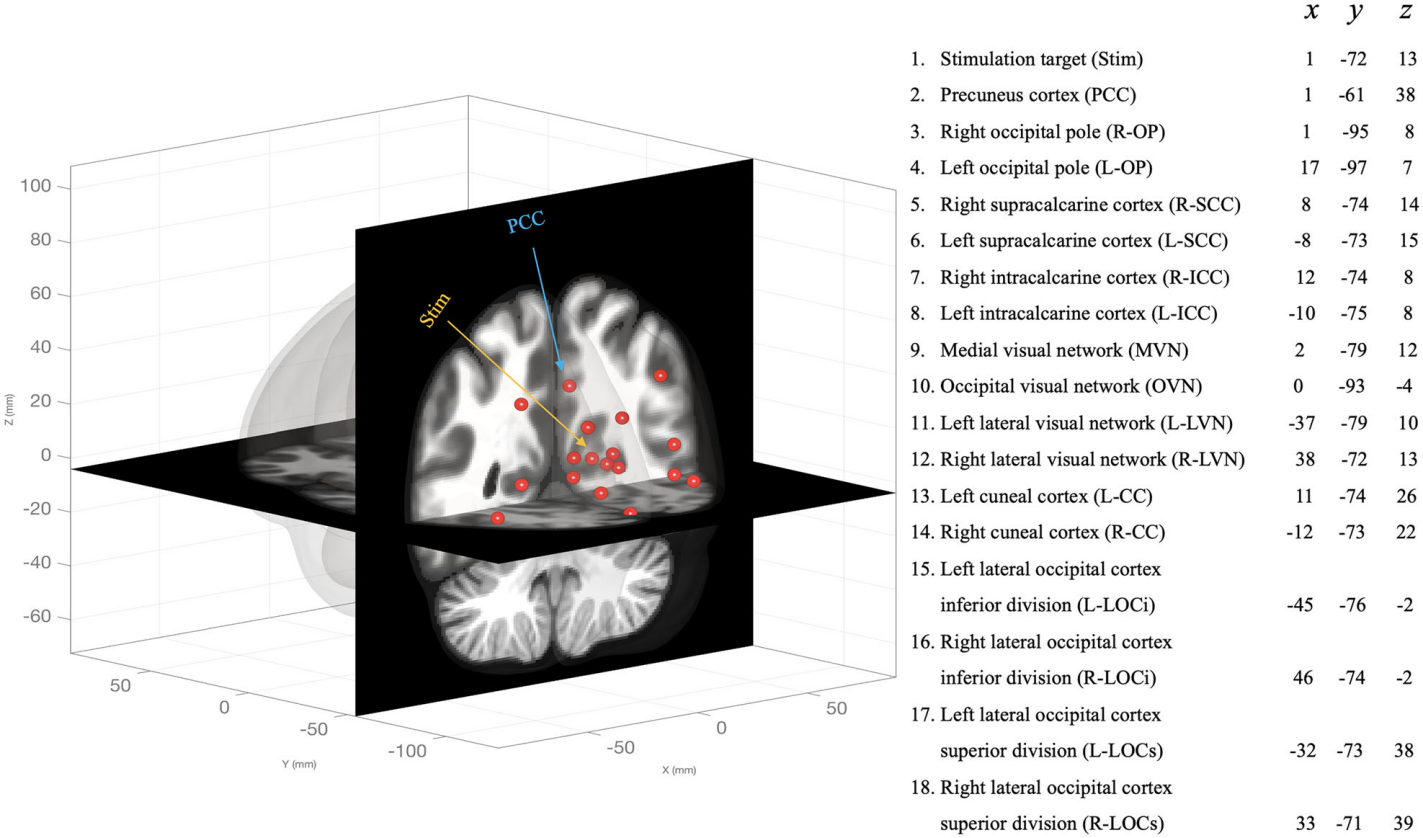
MNI coordinates of 18 ROIs used in ROI‐ROI analysis based on the Harvard‐Oxford atlas.

In addition to the whole brain FC analysis described above, we also examined the FC between 18 occipital and parietal ROIs in order to investigate post‐TBS changes in specific visual networks (see Figure 2). These ROIs were mainly chosen based on their proximities to the stimulation site seed ROI (covering bilateral focal and remote areas surrounding the stimulation ROI). The ROI‐to‐ROI connectivity matrices were computed with bivariate Pearson's correlation coefficients of BOLD signal for each pair of ROIs. The Pearson's correlation coefficients were then transformed to Fisher Z values for further statistical analyses. In order to correct for multiple comparisons, the cluster‐level *p* value was set at *p* < .05 (FDR‐corrected).

Lastly, we focused on the connections between the stimulation site and the 17 other chosen ROIs using a seed‐to‐target analysis. The 17 other ROIs were the same as the ROI‐to‐ROI analysis, but this approach is inherently different from the previous ROI‐to‐ROI analysis in that one seed was compared to the chosen targets (targets are not compared to one another), therefore requiring fewer comparisons to address a specific hypothesis. Using a mixed‐design ANOVA with FDR‐corrected *p* values < .05 we then examined between‐group differences in FC.

#### 2.8.2 Analysis 2: Surface‐based ROI‐to‐ROI analysis using GPIP parcellation

In this analysis, individual T1 images were parcellated into anatomical regions using the *recon‐all* pipeline in FreeSurfer v6.0.1 (http://surfer.nmr.mgh.harvard.edu/). Within FreeSurfer, the preprocessed and denoised time‐series output by ME‐ICA were coregistered to the T1‐weighted anatomical images output by recon‐all for each subject and session (bbregister). Next, the functional data were resampled to the fsaverage5 template image using trilinear volume‐to‐surface interpolation (mri_vol2surf). Spatial smoothing was applied using a full width half max kernel of 6 mm (mri_surf2surf). Using the surface space functional data, the time course of each resting state imaging run was normalized to a mean of 0 and a standard deviation of 1. Group Prior Individual Parcellation (GPIP; Chong et al., 2017) was used to output subject‐specific functional parcellations of the resting state data. Within GPIP, the data were first initialized using the 200‐parcel 7‐Network Schaefer atlas (Schaefer et al., 2018; for more information see Table S1 in Supplementary Materials) resulting in a common set of parcellation labels for all subjects. After initialization, GPIP uses each subject's resting state functional images to optimize the boundaries of each parcel, resulting in subject‐specific functional parcellations of resting state networks. The quality of GPIP parcellations for each subject was assessed by calculating the homogeneity of parcels at each of the 20 GPIP iterations. Homogeneity was calculated as the mean correlation coefficient of all pairs of vertices within each parcel and then averaged over all parcels in the brain for each subject to verify that these values increased over iterations and then plateaued in value prior to the final GPIP iteration.

**FIGURE 3:**
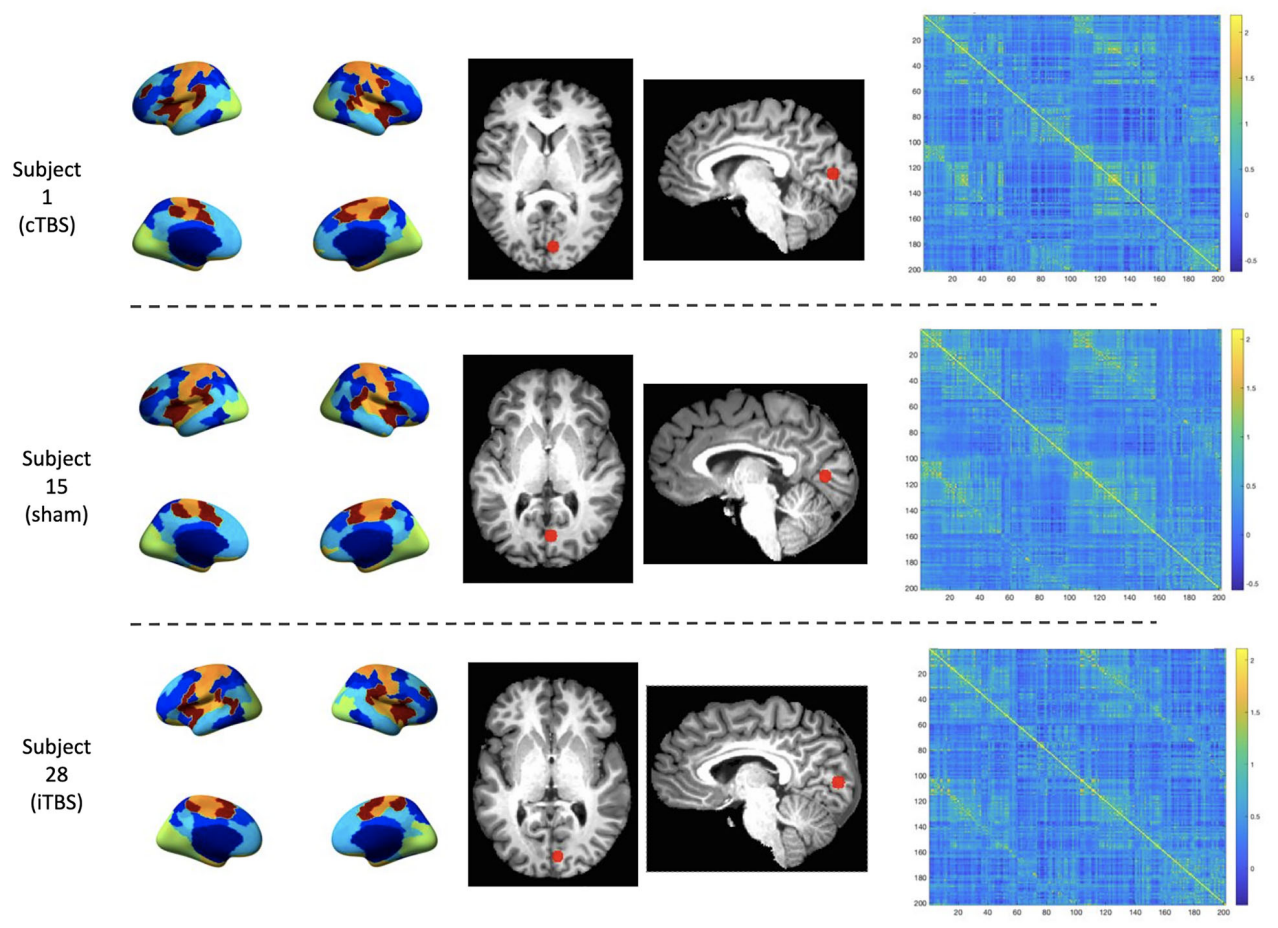
Example of individual parcellation and connectivity matrices in three randomly selected subjects from each of the three stimulation groups at baseline (day 1). cTBS = continuous theta burst stimulation; iTBS = intermittent theta burst stimulation; GPIP = Group Prior Individual Parcellation. The first two columns correspond to individual functional parcellations of each subject using GPIP. The middle two columns correspond to stimulation site ROIs in individual subject space, and the last column corresponds to the pairwise GPIP parcel (and stimulation site) Fisher Z connectivity values plotted as connectivity matrices for each subject.

Spherical ROI masks 5 mm in diameter were created for each subject/session using the coordinates of their stimulation site within V1 in AFNI. These ROI masks were then resampled to fsaverage5 surface space and inspected for accuracy of placement in each subject. The mean time‐series of each stimulation site ROI was extracted from the functional data (mri_segstats). Similarly, for each subject/session, the mean time‐series was extracted for each GPIP parcel. Pairwise Pearson's correlation coefficients were calculated for all extracted mean time‐series and then Fisher r‐to‐z transformed resulting in cross‐correlation functional connectivity matrices for each subject and session (see Figure 3 for examples of individual parcellation and connectivity matrices). Group analyses of parcel functional connectivity were performed using the Network Based Statistics toolbox (NBS; Zalesky et al., 2010). NBS V.2.0 (https://www.nitrc.org/projects/nbs) is a nonparametric method that controls the family‐wise error rate (FWER) when multivariate models are applied to neuroimaging data in order to compare functional or structural connectivity between pairs of ROIs or networks of ROIs. After setting up between‐ and within‐group contrasts and using the default FWER‐corrected significance level of 0.05, a whole brain FC analysis was first conducted. Using a 2 × 2 repeated measures ANOVA the Stimulation main effects were explored in each pair of conditions (e.g., cTBS > iTBS) across two timepoints (e.g., day 1 < day 2 immediately post‐TBS).

As the final step, and in order to choose comparable ROIs to the ones used in the volumetric analysis, the Schaefer 200‐parcel‐7‐network atlas that was used to initialize GPIP was overlayed on top of a standard MNI‐152 template in *fsleyes* (https://git.fmrib.ox.ac.uk/fsl/fsleyes/fsleyes/). Labels used in the Schaefer 200‐parcel 7‐network atlas correspond to resting state networks but do not correspond to specific anatomical region labels. Therefore, the Harvard‐Oxford cortical structural atlas was used to identify corresponding anatomical labels of the Schaefer atlas regions to facilitate comparison with the 17 ROIs used in the CONN Toolbox. Coordinates from the ROIs included in the volumetric analysis performed in CONN Toolbox and the homologous Schaefer atlas regions were manually determined and the connectivity values between the 17 selected GPIP ROIs and the stimulation site were extracted and compared (see Supplementary Table S1 for the list of ROIs). To examine FC within the visual networks we applied a less stringent correction method of FDR correction (< 0.05), and a series of 2 × 2 ANOVAs were conducted to determine the effects of Stimulation and Time on FC between groups at different timepoints. This procedure was performed using the visual network subset of the Schaefer 200‐parcel 7‐network atlas ROIs (28 ROIs plus the stimulation site; see Table S1 in Supplementary Materials).

## 3 RESULTS

### 3.1 Vision and cognitive assessments

For the visual acuity data (both monocular and binocular), and MoCA scores a two‐way mixed effects ANOVA revealed no significant interaction between Group (cTBS, iTBS, and sham) and Time (pre‐TBS and post‐TBS). Main effects of Group and Time were also nonsignificant (see Table 1 for group‐specific statistics).

**TABLE 1:** Analysis of variance (ANOVA) of phosphene thresholds, vision, and cognitive assessments data before and after TBS

		Pre‐TBS		Post‐TBS			ANOVA				
Assessment	Condition	*M*	*SD*	*M*	*SD*	Effect		*F* ratio	*df*	*p*	η^2^
PT (% Stimulator output)	cTBS	68.5	13.1	67.2	15.7	G		0.552	2, 27	.58	0.004
	iTBS	63.1	11.2	64.6	11.2	T		0.552	1, 27	.14	0.038
	Sham	60.1	13.7	64.2	10.7	G x T		2.69	2,27	.09	0.086
Binocular VA (LogMAR)	cTBS	−1.23	0.062	−0.108	0.051	G		0.167	2, 27	.85	0.011
	iTBS	−1.22	0.086	−0.146	0.077	T		0.221	1, 27	.64	0.001
	Sham	−0.13	0.11	−0.138	0.115	G x T		0.906	2, 27	.42	0.009
Right monocular VA (LogMAR)	cTBS	−0.043	0.063	−0.037	0.084	G		3.16	2, 27	.068	0.152
	iTBS	−0.078	0.051	−0.096	0.087	T		0.027	1, 27	.87	0.0002
	Sham	−0.13	0.105	−0.126	0.124	G x T		0.242	2, 27	.79	0.004
Left monocular VA (LogMAR)	cTBS	−0.089	0.07	−0.069	0.069	G		0.865	2, 27	.43	0.044
	iTBS	−0.115	0.07	−0.1	0.071	T		0.331	1, 27	.57	0.004
	Sham	−0.115	0.096	−0.123	0.112	G x T		0.283	2, 27	.76	0.006
MoCA	cTBS	27.9	1.60	27.8	1.48	G		0.019	2, 27	.98	0.001
	iTBS	27.9	0.99	28	1.25	T		0.672	1, 27	.42	0.006
	Sham	28.2	1.32	27.6	1.26	G x T		0.728	2, 27	.49	0.013

PT = phosphene threshold, TBS = theta burst stimulation, cTBS = continuous TBS, iTBS = intermittent TBS, VA = visual acuity, MoCA = Montreal Cognitive Assessment, LogMAR = logarithm of the minimum angle of resolution, Pre‐TBS = Day 1, before TBS, post‐TBS = Day 2, after TBS. Effect = G (Group: cTBS, iTBS, or sham), T (Time: pre‐ or post‐TBS), G x T (interaction between Group and Time). Significance level = *p* values < .05.

### 3.2 Phosphene thresholds

**FIGURE 4:**
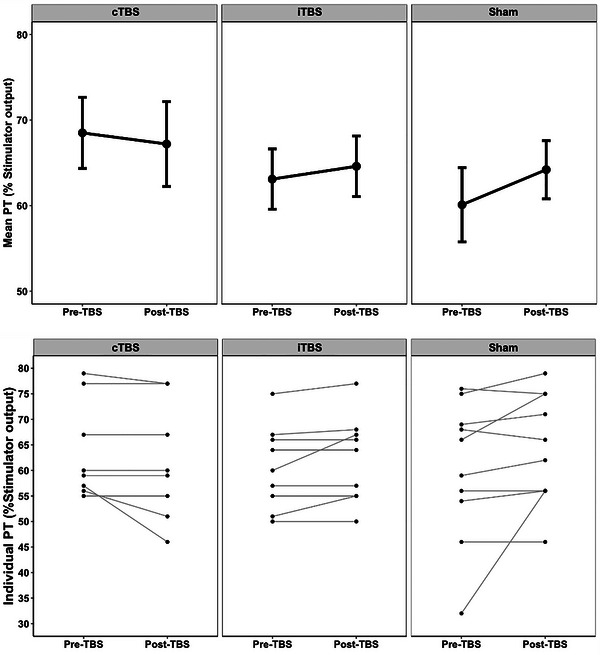
Pre‐ and post‐TBS group mean and individual phosphene thresholds. TBS = theta burst stimulation; cTBS = continuous TBS; iTBS = intermittent TBS. Pre = Day 1, pre‐TBS; Post = Day 2, 1 hr post‐TBS; PT = phosphene threshold, Stimulator output = TMS intensity required to evoke a phosphene.

As shown in Figure 4 (also see Table 1 for group‐specific descriptive and inferential statistics), the average pre‐ to post‐TBS PTs in all three groups did not change significantly. A two‐way mixed effects ANOVA found no interaction between Stimulation Group (cTBS, iTBS and sham) and Time (pre‐TBS and 1 h post‐TBS). There was no main effect of Stimulation Group.

### 3.3 Volumetric seed‐based connectivity analysis in MNI space

#### 3.3.1 Whole brain connectivity

An omnibus test to detect significant seed‐to‐voxel FC between conditions and timepoints was conducted. No significant interaction between Condition and Time was observed for whole brain connectivity. Similarly, there were no significant main effects of Group or Time for any of the seeds on whole brain connectivity.

#### 3.3.2 ROI‐to‐ROI

At the group level, we first examined individual connectivity for each condition (iTBS, cTBS, and sham) across the three timepoints. We used within‐group contrasts to determine the connectivity profile for each of the 18 chosen ROIs and 153 nonoverlapping connections. No significant interaction was found between Stimulation Condition and Time, and there were no significant main effects of Stimulation and Time on individual connectivity profiles.

#### 3.3.3 Seed‐to‐target

**FIGURE 5:**
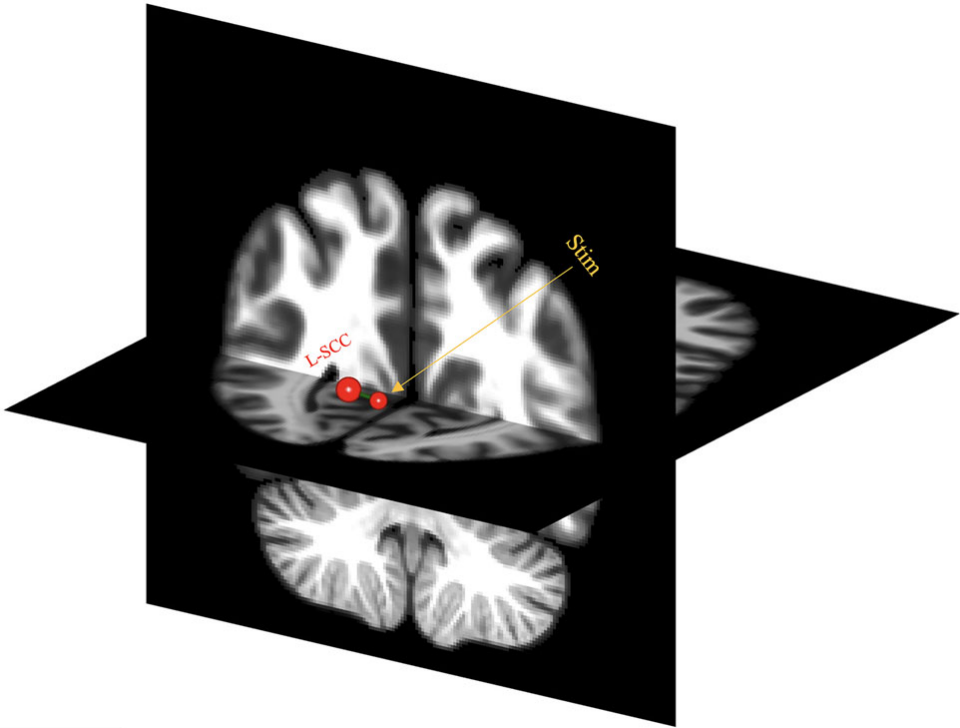
Seed‐to‐target analysis results. L‐SCC = Left supracalcarine cortex, Stim = stimulation site.

As shown in Figure 5, a between‐group analysis revealed one target ROI [left supracalcarine cortex (L‐SCC)] that survived the predefined threshold (*t* (18) = 3.59, *p*‐FDR = .036). However, pairwise comparisons revealed no significant difference in seed‐to‐target connectivity for the within‐condition (stimulation effect) at different timepoints (see Table S2 in Supplementary Materials for statistics).

### 3.4 Surface‐based ROI‐to‐ROI analysis using GPIP parcellation

#### 3.4.1 Whole brain FC

**FIGURE 6:**
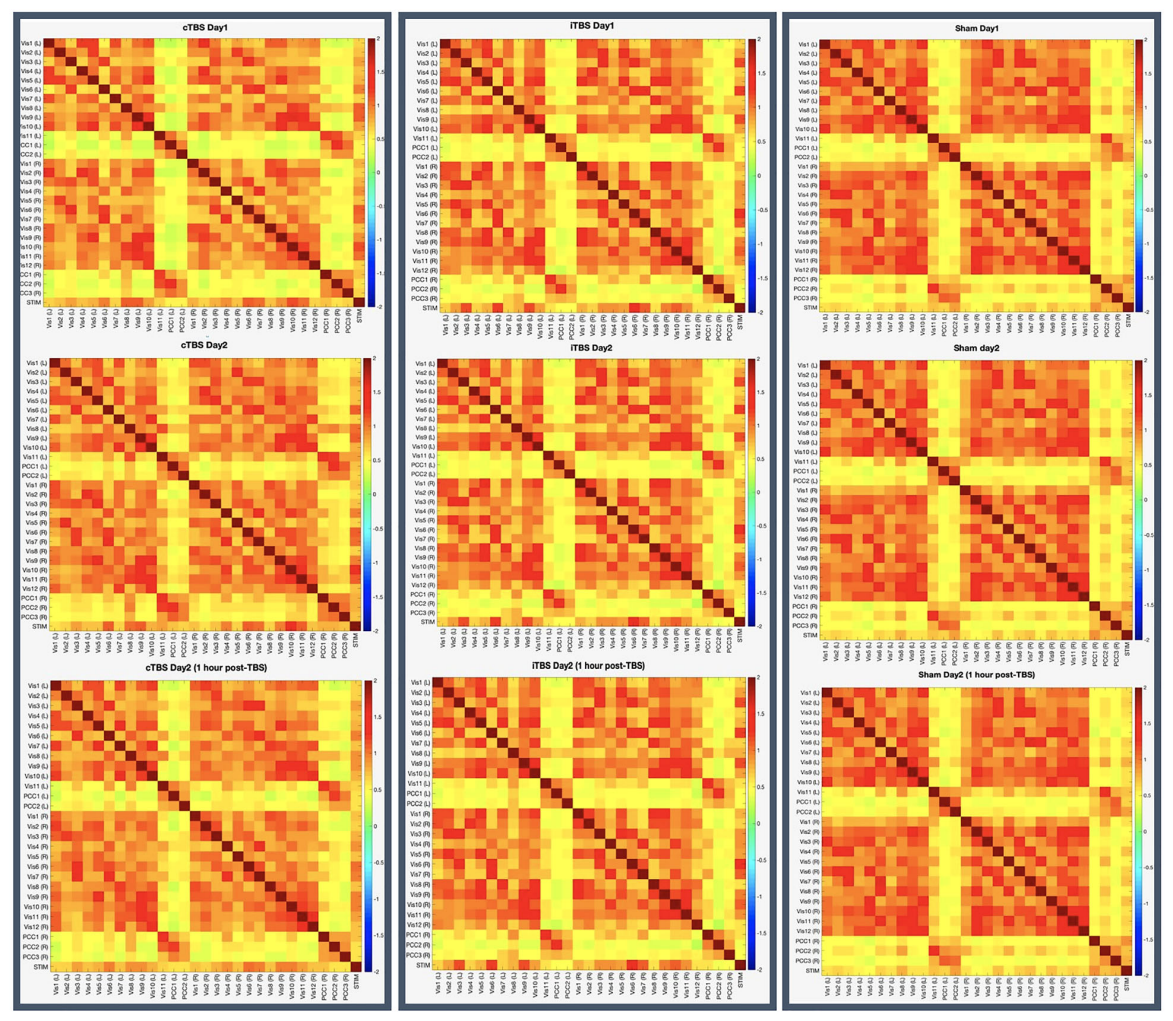
Uncorrected connectivity matrices using Fisher Z‐transformation of the Pearson's correlation coefficient values for each group and timepoint. Matrices include Schaefer 200‐parcel 7‐network atlas left and right hemisphere visual network regions, precuneus regions, and the stimulation site. For the list of ROIs and their coordinates see supplementary materials.

There were no significant differences in whole brain functional connectivity pre‐ and post‐stimulation (FWER‐corrected *p* values < .05). See Figure 6 for connectivity matrices.

#### 3.4.2 ROI to ROI and seed to target

There were no significant differences in functional connectivity for ROI‐to‐ROI and seed‐to‐target pre‐ to poststimulation (FDR‐corrected *p* value < .05 threshold was used).

## 4 DISCUSSION

In the current study, we determined the effects of TBS to primary visual cortex (V1) on visual cortex‐associated FC and phosphene thresholds (PTs). We compared group‐level FC across the entire brain and visual networks at three timepoints—baseline, immediately after stimulation and 1 h following stimulation. Despite employing different analysis methods to explore FC, we found no significant changes in FC pre‐ to post‐TBS. Additionally, we assessed PTs at baseline and 1 h following TBS and found no changes in cortical excitability levels post‐TBS. Overall, we did not identify any cTBS or iTBS related aftereffects on FC and PTs. These results are consistent with our MRS study of the same cohort using the same experimental design, where cTBS and iTBS did not alter the concentration levels of GABA and glutamate at the stimulation site (see Stoby et al., 2022).

The fact that a single session of cTBS and iTBS had no effect on FC does not refute the efficacy of TBS to V1, however, it may very well highlight the challenges in determining the optimal stimulation dosage for the use of TBS to modulate FC associated with the visual cortex in clinical settings. The transient effects of traditional TMS protocols to visual regions of the brain have been reported for years (Ganaden et al., 2013; Mullin & Steeves, 2013; Solomon‐Harris et al., 2016). In addition, our previous TMS‐fMRI study at visual cortex using a low frequency (1 Hz) rTMS paradigm found widespread FC changes 1 h following a single session of rTMS (Rafique & Steeves, 2022). These contrasting findings may suggest that TBS is not a suitable replacement for traditional rTMS when stimulating V1 since it does not yield equivalent effects. It may be that the shorter TBS stimulation time is insufficient to reach optimal thresholds required to disturb synaptic equilibrium and neuronal status quo in order to detect TBS aftereffects with rs‐fMRI. One possible mechanism to consider is the differential abilities of TBS and rTMS protocols to entrain oscillations in targeted neural populations. Oscillations have long been studied and implicated as location‐ and state‐dependent neural signatures of the central nervous system (Buzsáki, 2004). Recent literature suggests that stimulation parameters (i.e., oscillations resembling specific brain rhythms) closer to the specific intrinsic oscillatory patterns of the stimulated location can lead to entrainment and therefore more effective stimulation outcomes (Lin et al., 2021; Okazaki et al., 2021; Thut et al., 2011). For instance, previous studies using electro‐ and magneto‐encephalography have shown that during memory and motor sequence learning tasks theta oscillations (∼ 6–7 Hz) are the predominant recorded oscillations at the scalp electrodes closest to hippocampus and motor cortex (Meissner et al., 2018; Tesche & Karhu, 2000). Execution of already mastered motor tasks, however, is correlated with beta oscillations (∼ 13–30 Hz) recorded at motor cortex (Baker, 2007; Pfurtscheller & Lopes da Silva et al., 1999). In addition, gamma oscillations (∼ 30–50 Hz) have been recorded during high‐order cognitive tasks in temporal and frontal brain regions (Buzsáki & Wang, 2012; Singh et al., 2020), and intrinsic occipital alpha oscillations (∼ 8–12 Hz) have been linked to the perception of incoming visual stimuli (e.g., eyes open and closed) and attending to such inputs (Ergenoglu et al., 2004). TMS studies in V1 have been able to validate the notion of synchronicity between TMS and intrinsic brain rhythms by entraining occipital alpha oscillations via a 10 Hz rTMS protocol (Lin et al., 2021; Romei et al., 2010). Theta oscillations, however, were the basic elements upon which TBS protocols have been developed (Larson et al., 1986), and as theta oscillations are mainly recorded in frontal and motor regions, TBS may better target and manipulate M1 and frontal regions. Therefore, such oscillatory properties may also explain the efficacy of cTBS and iTBS in motor and frontal cortices but not in V1.

In addition, our single session of TBS to V1 at 80% PT did not produce measurable effects in rs‐fMRI which is consistent with a study by Rahnev et al. (2013) where cTBS, iTBS and sham TBS were applied to the scalps of five subjects at V1 using a stimulation intensity of 80% PT followed by rs‐fMRI. They also found that iTBS did not have a significant effect on FC; they did, however, find a significant decrease in FC between retinotopically defined early visual areas (i.e., V1, V2, and V3) following cTBS. Similarly, others have observed no change in PTs following iTBS but a reduction in PTs measured 2 min post‐cTBS (80% PT, 600 pulses; Franca et al., 2006). Other TBS studies, however, have adopted different dosing strategies using lower intensities and increased pulse numbers. For example, when cTBS targeted the right occipital face area and the posterior superior temporal sulcus (pSTS) at 80% active MT (or 30% maximum stimulator output; whichever was greater) delivering 900 pulses, cTBS reduced BOLD signal in face selective areas (Pitcher et al., 2014) and reduced FC between pSTS and amygdala (Pitcher et al., 2014b, 2017). Other studies using the same low intensity and high number of pulses as the stimulation parameters demonstrated that cTBS reduced FC between pSTS and other vision and nonvision ROIs (Handwerker et al., 2020) and reduced BOLD signal in the occipital place area (Groen et al., 2021). This stimulation protocol may support the efficacy of cTBS in yielding inhibitory effects in visual areas, however, other TBS studies such as Abuleil et al. (2021) have shown that cTBS to V1 increases mixed percepts during binocular rivalry (excitatory effect).

Occipital TBS studies have used a variety of dosing strategies ranging from lower intensities to increased pulse numbers, and despite clear anatomical and functional distinctions between M1 and V1 (Shinomoto et al., 2009) a number of studies utilized active MTs to determine optimal TBS intensities. Previously, four studies had compared MTs (resting and active) and PTs and concluded that these two measures are not correlated, and that PTs are the most accurate measure of cortical excitability in the occipital cortex and MTs should not be utilized for nonmotor TMS targets (Antal et al., 2003; Boroojerdi et al., 2002; Gerwig et al., 2003; Stewart et al., 2001). Nevertheless, Deblieck et al. (2007) demonstrated a significant correlation between active MTs and PTs, and the notion of a universal cortical excitability value (based on MTs) appears to dominate TBS research in frontal, parietal and temporal regions and to some extent in the occipital visual areas (Stokes et al., 2013). However, it remains unclear whether the two thresholds (MT and PT) are comparable measures of cortical excitability, given the design and methodological limitations of MT‐PT studies, and the anatomical and functional differences between M1 and V1. It is therefore plausible to speculate that whether the individual cortical excitability in V1 is measured via MTs or PTs, subthreshold intensities and increased pulse number may play a role in cTBS outcomes (Groen et al., 2021; Handwerker et al., 2020; Pitcher et al., 2014b, 2017). Knowing that on average MTs are lower than PTs (Boroojerdi et al., 2002), a 30% MT translates into a much lower stimulation intensity than intensities delivered at 80% PT. As a result, in comparison to other occipital TBS studies (using 80% PT intensity such as Franca et al., 2006 and Rahnev et al., 2013), these intensities are considered subthreshold.

Modifying the number of pulses in a stimulation protocol also can modulate TBS effects. The respective inhibitory and excitatory effects of cTBS and iTBS in M1 first observed by Huang et al. (2005) are reversed by doubling the number of pulses. At 1200 pulses for example, cTBS increased MEP amplitudes while iTBS lowered MEP amplitudes (Gamboa et al., 2010). It is possible that the 900‐pulse protocol (e.g., Pitcher et al., 2014b, 2017) allows for an increased pulse delivery without reversing the inhibitory effects of cTBS when targeting visual networks. However, at the moment, comparison studies examining different TBS parameters in occipital regions are lacking, and despite a vast literature exploring pulse numbers, sub‐ and supra‐threshold stimulation intensities, and accelerated TBS protocols in frontal lobes (Chen et al., 2021; Lee et al., 2021; McCalley et al., 2021) only one occipital TBS study has examined the effects of TBS intensities on visual perception (and not on PTs). When cTBS and iTBS were applied to V1 using stimulation intensities of 60, 80,100 and 120% PT, both cTBS and iTBS had no effect on peripheral visual acuity at any of the intensity levels (Brückner & Kammer, 2014).

In addition to the variability of TBS parameters, outcome variability in TBS research may be modulated by a high individual variability in stimulation responsiveness (McCalley et al., 2021; Young‐Bernier et al., 2014), leading to a statistical net‐zero effect when subjects with variable responses to cTBS or iTBS are pooled in a group and outcome measures are averaged. In the present study, we also observed this pattern of variability in our FC analyses in both the cTBS and iTBS groups. To mitigate the effects of individual variability on stimulation outcomes, Ridding and Ziemann (2010) had previously identified a list of contributing factors influencing TMS outcomes. Factors such as diurnal cortisol levels, age, attention, synaptic history (pharmacological agents, and prior stimulation), and genetics were all identified to affect the stimulation outcomes to varying degrees. Although many studies including our current study have controlled for these factors, genetic variations also seem to play an important role in neural response to stimulation (Cheeran et al., 2008). Brain‐derived neurotrophic factor (BDNF) is a protein that reportedly is involved in LTP and LTD processes. It is speculated that BDNF directly affects the susceptibility of synapses to undergo plasticity and change in response to learning and stimulation (Lu, 2003). Genetic polymorphism in BDNF expression factors such as the heterozygous genotype “val66met” has been linked to decreased response to noninvasive brain stimulation protocols at M1 unlike homozygous Met/Met and Val/Val carriers (Cheeran et al., 2008; Fritsch et al., 2010; Guerra et al., 2020).

In summary, our findings show no effects of cTBS or iTBS to V1 on FC across visual networks nor any effects on PTs. These findings, together with the paucity of research on TBS to V1 and the heterogeneity of protocols used in other visual regions suggest that further research is needed to refine the protocols for optimal TBS dosage in visual networks. This is especially important because the traditional rTMS studies showing lasting aftereffects generally used much longer stimulation duration, while TBS protocols have a much shorter stimulation duration. Despite the fact that multiple studies have implied the comparability of iTBS and 10 Hz rTMS in major depressive disorders (Blumberger et al., 2018) as well as cTBS and 1 Hz rTMS in frontoparietal regions (Tupak et al., 2011; Yu‐Lei et al., 2022), such comparisons have yet to be determined in TBS to V1 or occipital cortex more broadly.

While our study did not reveal any significant effects of cTBS and iTBS to V1 in healthy individuals, the potential of noninvasive brain stimulation to improve therapeutic outcomes in patients with neurological visual disorders is still a promising area of research. Our findings contribute to the growing body of literature in this field, reporting null findings can be informative in designing future studies as it helps to rule out alternative hypotheses and refines our understanding of different TBS protocols and methodologies. Furthermore, our study underscores the need for further investigation into optimal TBS dosage by studying different stimulation parameters. As discussed in detail earlier in this section, it is crucial to study the root cause of interindividual variability in TBS outcomes by examining response variations in healthy subjects, while considering factors such as BDNF expression and the neural entrainment properties of different rTMS protocols at specific target locations. We believe that combining insights from a growing number of TBS studies in visual brain areas, regardless of their outcomes, will open up exciting avenues of research in the near future.

## 5 LIMITATIONS AND FUTURE RESEARCH

In the present study, measuring PTs immediately after TBS could not have been achieved, mainly due to rs‐fMRI timepoints (immediately and 1 h post‐TBS). Determining PTs requires stimulation, which would have prevented us from measuring the true aftereffects of TBS on resting state networks at 1 h. We therefore opted for measuring PTs at 1 h (after the last rs‐fMRI scan). Despite this limitation, however, these results could be taken into consideration when designing future experiments. On the one hand, the PT results could be interpreted as the evidence that TBS cannot modify PTs when measured 1 h after stimulation. On the other hand, this may suggest that if cTBS or iTBS are capable of inducing directional changes to PTs, these effects wear off after 1 h and thresholds return to baseline. This could indicate that experiments utilizing within‐subjects designs can consider using this 1 h cut‐off when determining optimal “rest periods” required for participants undergoing multisession TBS protocols (e.g., comparison studies).

As we highlighted in the discussion, future research should focus on optimization of TBS protocols in V1 by designing studies that compare the ability of different TBS protocols to influence intrinsic oscillatory processes at different brain areas (e.g., M1 vs. V1). Despite a vast number of comparison studies in M1 and nonmotor frontal regions, parameters such as pulse number, stimulation intensities, and accelerated TBS protocols should also be explored in V1 while taking covariates such as genetic polymorphism (i.e., BDNF expression factors) and anatomical differences (e.g., scalp to cortex distance and target depth) into account.

## 6 CONCLUSION

In the current study, we demonstrate that the application of cTBS and iTBS to V1 does not modulate resting state FC in focal or remote brain networks when measured immediately and 1 h post‐TBS. PT levels also remain unaffected by cTBS and iTBS when measured 1 h after stimulation. These results are consistent with our MRS study, where cTBS and iTBS did not alter GABA and glutamate concentration levels (Stoby et al., 2022). Our results are also in line with others who showed iTBS did not modulate FC and PT levels. Our findings show that while cTBS and 1 Hz rTMS may have comparable effects at the motor and frontal cortices (Blumberger et al., 2018; Tupak et al., 2011; Yu‐Lei et al., 2022) cTBS to V1 does not have comparable effects to low frequency (1 Hz) rTMS in our previous study (Rafique & Steeves, 2022) .

Our findings suggest that in a clinical setting, a single session of cTBS or iTBS to V1 at 80% PT using a protocol of 600 pulses may not be an effective therapy if targeting FC is the clinical goal.

### PEER REVIEW

The peer review history for this article is available at https://publons.com/publon/10.1002/brb3.2989.

## Supporting information

Supp Information

## Data Availability

The data that support the findings of this study are available from the corresponding author upon reasonable request.
