# Supplementary material for: Continuous and intermittent theta burst stimulation of primary visual cortex do not modulate resting state functional connectivity: A sham‐controlled multi‐echo fMRI study
Source: Brain Behav. 2023 Apr 16;13(5):e2989. doi: 10.1002/brb3.2989 (PMC10175989; doi:10.1002/brb3.2989)
Supplement: Supplementary file 1 — Supp Information [file BRB3-13-e2989-s001.docx]

# **Supplementary materials**

**Table S1**

*Schaefer-200 node conversion to MNI coordinates.*

|  | **MNI Coordinates** | | | |  |
| --- | --- | --- | --- | --- | --- |
| **Schaefer Atlas** | | ***x*** | ***y*** | ***z*** | **Corresponding MNI region** |
| Vis1 (L) | | -24 | -53 | -9 | 51% lingual gyrus, 33% temporal occipital fusiform cortex |
| Vis2 (L) | | -26 | -77 | -14 | 64% occipital fusiform gyrus, 6% lingual gyrus |
| Vis3 (L) | | -45 | -69 | -8 | 71 % lateral occipital cortex |
| Vis4 (L) | | -10 | -67 | -4 | 67% lingual gyrus, 6% occipital fusiform gyrus |
| Vis5 (L) | | -27 | -95 | -12 | 55 % occipital pole, 10% lateral occipital cortex (inferior division) |
| Vis6 (L) | | -14 | -44 | -3 | 40 % lingual gyrus, 30 % cingulate gyrus posterior division, |
| Vis7 (L) | | -5 | -93 | -4 | 62 % occipital pole, 11% lingual gyrus, 11 % Intracalcarine cortex |
| Vis8 (L) | | -47 | -70 | 10 | 66% lateral occipital |
| Vis9 (L) | | -23 | -97 | 6 | 56% occipital pole |
| Vis10 (L) | | -11 | -70 | 7 | 55% intracalcarine cortex, 10% lingual gyrus |
| Vis11 (L) | | -40 | -85 | 11 | 36 % lateral occipital cortex superior division, 32% lateral occipital inferior division |
| PCC1 (L) | | -11 | -56 | 13 | 46% precuneus cortex, |
| PCC2 (L) | | -6 | -54 | 42 | 75% precuneus cortex |
| Vis1 (R) | | 39 | -35 | -23 | 58% lingual gyrus, 30% occipital fusiform |
| Vis2 (R) | | 28 | -36 | -14 | 51% lingual gyri |
| Vis3 (R) | | 29 | -69 | -12 | 53 % occipital fusiform gyrus, 8% lingual gyrus |
| Vis4 (R) | | 12 | -65 | -5 | 68% lingual gyrus, 8% occipital fusiform gyrus |
| Vis5 (R) | | 48 | -71 | -6 | 68% lateral occipital cortex inferior division |
| Vis6 (R) | | 11 | -92 | -5 | 51% occipital pole, 10 lingual gyrus, 5% occipital fusiform gyrus |
| Vis7 (R) | | 16 | -46 | -1 | 48% lingual gyrus, 37% cingulate gyrus posterior division |
| Vis8 (R) | | 31 | -94 | -4 | 64% occipital pole, 6% lateral occipital cortex inferior division |
| Vis9 (R) | | 9 | -75 | 9 | 64% intracalcarine cortex, 6% supracalcarine cortex, |
| Vis10 (R) | | 22 | -60 | 7 | 37 % Intracalcarine cortex, 36% precuneus cortex |
| Vis11 (R) | | 42 | -80 | 10 | 52% lateral occipital cortex inferior division, 29% lateral occipital superior cortex |
| Vis12 (R) | | 20 | -90 | 22 | 31% occipital pole, 10% lateral occipital cortex superior division |
| PCC1 (R) | | 12 | -55 | 15 | 60% precuneus cortex, 8% supracalcarine cortex |
| PCC2 (R) | | 7 | -49 | 31 | 58% cingulate gyrus posterior division, 12% precuneus cortex |
| PCC3 (R) | | 6 | -58 | 44 | 73% precuneus cortex |
| Stimulation site | | 1 | -72 | 13 | 46% supracalcarine cortex, 23% Intracalcarine cortex |

*Note*. ROIs in black and red were all included in ROI-to-ROI analysis. Only the ROIs in red were included in the seed-to-target analysis. 200-parcel 7-Network Schaefer atlas includes 1) Visual 2) Somatomotor 3) Dorsal attention 4) Ventral attention 5) Limbic 6) Frontoparietal 7) Default mode networks (Schaefer et al., 2018)

**Table S2**

*Results of the rs-fMRI seed-to-target data analysis at three timepoints (Day 1 pre-TBS, Day 2 immediately post-TBS and Day 2 1 hr post-TBS).*

| Seed: Stimulation site | | | | | | |
| --- | --- | --- | --- | --- | --- | --- |
| Contrast | | Day 2 (immediately post-TBS) > Day1 | | | | |
|  | |  | *beta* | *t* (18) | *p*-unc | *p*-FDR |
| iTBS > cTBS | | PCC  R-OP  L-OP  R-SCC  L-SCC  R-ICC  L-ICC  MVN  OVN  L-LVN  R-LVN  L-CC  R-CC  L-LOCi  R-LOCi  L-LOCs  R-LOCs | 0.02  0.02  0.02  0.03  0.03  0.08  0.03  0.02  0.05  -0.04  -0.11  0.00  -0.06  -0.02  -0.02  -0.06  -0.20 | 0.26  0.22  0.15  0.39  0.33  0.71  0.33  0.20  0.45  -0.48  -1.10  -0.03  -0.46  -0.15  -0.21  -0.71  -2.07 | 0.80  0.83  0.88  0.69  0.75  0.49  0.75  0.84  0.66  0.64  0.29  0.98  0.65  0.88  0.83  0.49  0.05 | 0.98  0.98  0.98  0.98  0.98  0.98  0.98  0.98  0.98  0.98  0.98  0.98  0.98  0.98  0.98  0.98  0.90 |
| Sham > cTBS | | PCC  R-OP  L-OP  R-SCC  L-SCC  R-ICC  L-ICC  MVN  OVN  L-LVN  R-LVN  L-CC  R-CC  L-LOCi  R-LOCi  L-LOCs  R-LOCs | -0.06  0.03  0.05  0.01  0.04  0.05  0.05  0.07  -0.01  0.03  -0.07  0.12  0.03  0.04  0.07  0.01  -0.23 | -0.79  0.32  0.46  1.25  0.59  0.57  0.59  0.85  -0.08  0.38  -0.60  1.21  0.34  0.49  0.63  0.17  -2.64 | 0.44  0.75  0.65  0.23  0.57  0.57  0.56  0.41  0.94  0.71  0.56  0.24  0.73  0.63  0.53  0.87  0.02 | 0.85  0.85  0.85  0.85  0.85  0.85  0.85  0.85  0.94  0.85  0.85  0.85  0.85  0.85  0.85  0.85  0.28 |
| iTBS > Sham | | PCC  R-OP  L-OP  R-SCC  L-SCC  R-ICC  L-ICC  MVN  OVN  L-LVN  R-LVN  L-CC  R-CC  L-LOCi  R-LOCi  L-LOCs  R-LOCs | 0.09  -0.01  -0.03  -0.08  -0.04  0.03  -0.02  -0.06  0.06  -0.08  -0.04  -0.12  -0.09  -0.06  -0.09  -0.06  0.03 | 1.1  -0.10  -0.27  -0.98  -0.45  0.21  -0.22  -0.50  0.53  -0.98  -0.50  -1.25  -0.79  -0.71  -0.91  -0.71  0.49 | 0.22  0.92  0.79  0.34  0.67  0.83  0.84  0.63  0.61  0.34  0.62  0.23  0.44  0.49  0.38  0.49  0.62 | 0.86  0.92  0.89  0.86  0.86  0.89  0.89  0.87  0.86  0.86  0.86  0.86  0.86  0.86  0.86  0.86  0.86 |
|  | |  |  |  |  |  |
| Contrast | Day 2 (1 hr post-TBS) > Day1 | | | | | |
|  | | Target | *beta* | *t* (18) | *p*-unc | *p*-FDR |
| iTBS > cTBS | | PCC  R-OP  L-OP  R-SCC  L-SCC  R-ICC  L-ICC  MVN  OVN  L-LVN  R-LVN  L-CC  R-CC  L-LOCi  R-LOCi  L-LOCs  R-LOCs | 0.01  -0.02  0.01  0.08  0.30  0.04  0.14  0.02  0.02  -0.03  -0.01  0.08  0.06  0.02  0.08  -0.03  -0.10 | 0.15  -0.15  0.14  0.55  3.05  0.32  10.3  0.18  0.23  -0.34  -0.12  0.70  0.55  0.23  0.82  -0.37  -1.47 | 0.90  0.88  0.89  0.59  0.007  0.75  0.32  0.86  0.82  0.74  0.91  0.50  0.60  0.82  0.42  0.72  0.16 | 0.91  0.91  0.91  0.91  0.12  0.91  0.91  0.91  0.91  0.91  0.91  0.91  0.91  0.91  0.91  0.91  0.91 |
| Sham > cTBS | | PCC  R-OP  L-OP  R-SCC  L-SCC  R-ICC  L-ICC  MVN  OVN  L-LVN  R-LVN  L-CC  R-CC  L-LOCi  R-LOCi  L-LOCs  R-LOCs | -0.07  0.05  0.11  0.08  0.12  0.02  0.12  -0.03  0.08  0.10  0.05  -0.02  -0.06  0.25  0.12  0.15  -0.08 | -0.74  0.42  0.95  0.70  1.52  0.15  0.89  -0.25  0.56  0.88  0.43  -0.24  -0.49  1.23  1.03  1.23  1.22 | 0.47  0.68  0.35  0.50  0.14  0.88  0.38  0.80  0.59  0.39  0.68  0.83  0.63  0.23  0.32  0.24  0.24 | 0.82  0.82  0.82  0.82  0.82  0.88  0.82  0.86  0.82  0.82  0.82  0.82  0.82  0.82  0.82  0.82  0.82 |
| iTBS > Sham | | PCC  R-OP  L-OP  R-SCC  L-SCC  R-ICC  L-ICC  MVN  OVN  L-LVN  R-LVN  L-CC  R-CC  L-LOCi  R-LOCi  L-LOCs  R-LOCs | 0.05  -0.02  -0.03  0.01  0.19  0.00  0.05  -0.04  0.03  -0.02  0.00  0.04  0.02  0.03  0.05  -0.03  0.01 | 1.15  -0.23  -0.33  0.15  3.01  0.00  0.75  -0.45  0.31  -0.18  0.05  0.54  0.32  0.31  0.57  -0.71  0.10 | 0.27  0.82  0.75  0.88  0.007  0.99  0.46  0.66  0.76  0.86  0.96  0.59  0.75  0.76  0.57  0.48  0.92 | 0.99  0.99  0.99  0.99  0.13  0.99  0.99  0.99  0.99  0.99  0.99  0.99  0.99  0.99  0.99  0.99  0.99 |
|  | |  |  |  |  |  |
| Contrast | Day 2 (1 hr post-TBS) > Day2 (immediately post-TBS) | | | | | |
|  | | Target | *beta* | *t* (18) | *p*-unc | *p*-FDR |

| iTBS > cTBS | PCC  R-OP  L-OP  R-SCC  L-SCC  R-ICC  L-ICC  MVN  OVN  L-LVN  R-LVN  L-CC  R-CC  L-LOCi  R-LOCi  L-LOCs  R-LOCs | -0.01  -0.04  0.00  0.05  0.30  -0.04  0.11  0.00  -0.02  0.01  0.01  0.08  0.12  0.04  0.11  0.03  0.09 | -0.15  -0.29  -0.01  0.46  3.59  -0.28  0.87  0.04  -0.16  0.13  0.73  0.76  1.00  0.45  0.99  0.27  0.85 | 0.88  0.77  0.99  0.65  0.002  0.78  0.40  0.97  0.88  0.89  0.47  0.45  0.33  0.65  0.34  0.79  0.41 | 0.99  0.99  0.99  0.99  0.036  0.99  0.99  0.99  0.99  0.99  0.99  0.99  0.99  0.99  0.99  0.99  0.99 |
| --- | --- | --- | --- | --- | --- |
| Sham > cTBS | PCC  R-OP  L-OP  R-SCC  L-SCC  R-ICC  L-ICC  MVN  OVN  L-LVN  R-LVN  L-CC  R-CC  L-LOCi  R-LOCi  L-LOCs  R-LOCs | 0.00  -0.02  -0.08  -0.02  0.08  -0.03  0.06  -0.11  0.09  0.07  0.12  -0.14  -0.09  0.10  0.05  0.03  0.15 | -0.05  -0.20  0.66  -0.20  0.97  -0.33  0.49  -1.29  0.62  0.59  0.88  -1.92  -1.19  0.96  0.49  0.34  1.58 | 0.96  0.84  0.52  0.35  0.35  0.75  0.63  0.21  0.54  0.56  0.39  0.071  0.25  0.35  0.74  0.74  0.13 | 0.96  0.96  0.90  0.96  0.90  0.90  0.90  0.90  0.90  0.90  0.90  0.90  0.90  0.90  0.90  0.91  0.90 |
| iTBS > Sham | PCC  R-OP  L-OP  R-SCC  L-SCC  R-ICC  L-ICC  MVN  OVN  L-LVN  R-LVN  L-CC  R-CC  L-LOCi  R-LOCi  L-LOCs  R-LOCs | -0.01  -0.06  -0.07  0.07  0.22  0.00  0.05  0.11  -0.11  -0.06  -0.02  0.22  0.21  -0.07  0.05  -0.01  -0.06 | -0.11  -0.73  -0.79  0.70  3.03  -0.04  0.42  1.02  -1.15  -0.57  -0.14  2.04  1.89  -0.74  0.55  -0.10  -0.71 | 0.91  0.47  0.44  0.49  0.007  0.97  0.68  0.32  0.26  0.57  0.89  0.056  0.076  0.47  0.59  0.92  0.49 | 0.97  0.83  0.83  0.83  0.12  0.97  0.90  0.83  0.83  0.83  0.97  0.43  0.43  0.83  0.83  0.97  0.83 |

*Note*. For the full list of ROIs (and their acronyms) refer to Figure 2. The only significant connection between the stimulation site and L-SCC is highlighted in red. p-unc = ROI-level uncorrected p values set at < 0.05, p-FDR = ROI-level false discovery rate corrected p-value set at < 0.05. Beta values represent the effect size based on Fisher-Z transformed correlation values.
